# Correlation between Renal Function and Common Risk Factors for Chronic Kidney Disease in a Healthy Middle-Aged Population: A Prospective Observational 2-Year Study

**DOI:** 10.1371/journal.pone.0113263

**Published:** 2014-11-14

**Authors:** Michiya Ohno, Fumiko Deguchi, Kumiko Izumi, Hirotoshi Ishigaki, Hiroshi Sarui, Akihiko Sasaki, Tomonori Segawa, Takahiko Yamaki, Takao Kojima, Hiroshige Ohashi

**Affiliations:** 1 Division of Nephrology, Murakami Memorial Hospital, Asahi University School of Dentistry, Gifu City, Gifu, Japan; 2 Division of Health Center, Murakami Memorial Hospital, Asahi University School of Dentistry, Gifu City, Gifu, Japan; 3 Division of Diabetes and Endocrinology, Murakami Memorial Hospital, Asahi University School of Dentistry, Gifu City, Gifu, Japan; 4 Division of Cardiology, Murakami Memorial Hospital, Asahi University School of Dentistry, Gifu City, Gifu, Japan; Mario Negri Institute for Pharmacological Research and Azienda Ospedaliera Ospedali Riuniti di Bergamo, Italy

## Abstract

**Background/Aims:**

Age, proteinuria, metabolic syndrome, and hyperuricemia are the reported risk factors for chronic kidney disease (CKD) and cardiovascular disease (CVD). However, the best predictor of changes in renal function in the early stages of renal disease in a healthy middle-aged population is still unknown. Our study evaluated the correlation between changes in renal function and common risk factors to determine such a predictor.

**Methods:**

In total, 2,853 healthy persons aged ≤50 years participated in the study. They had no proteinuria and were not on medications for hypertension, diabetes mellitus, hyperlipidemia, or hyperuricemia. Over 2 years, participants underwent annual health screening. The relationship between changes in estimated glomerular filtration rate (eGFR) and changes in risk factors for CKD was evaluated using univariate and multivariate linear regression analyses.

**Results:**

Over 2 years, eGFR showed a significant decrease. Univariate regression analysis revealed that changes in fasting plasma glucose (FPG), total cholesterol, LDL-cholesterol, serum uric acid levels, and hemoglobin showed a significant negative correlation with changes in eGFR. Multiple regression analysis confirmed that changes in FPG, serum uric acid levels, in particular, and hemoglobin had a significant negative correlation with changes in eGFR.

**Conclusion:**

The changes in eGFR and other variables over 2 years were small and could be within expected biologic variation. A longer observational study is needed to elucidate whether FPG, serum uric acid and hemoglobin represent the earliest markers of eGFR decline.

## Introduction

Chronic kidney disease (CKD) increases the risk of end-stage renal disease and cardiovascular disease (CVD) [1]–[3]. In Japan, the prevalence of CKD is approximately 13% of the adult population [4]. CKD is classified into stages 1 to 5 based on the levels of proteinuria and estimated glomerular filtration rate (eGFR), which is calculated from the age and serum creatinine level [5]. As CKD progresses through these 5 stages, the risk of CVD increases; CVD associated with CKD is known as the cardiorenal syndrome [1]–[3]. CKD and CVD also share common risk factors, such as hypertension, diabetes mellitus, and dyslipidemia [6]. Recently, lifestyle-related metabolic syndrome and hyperuricemia have also been reported as risk factors for CKD [7]–[10].

The most significant factor contributing to renal function decline in healthy persons is aging; however, significant individual variation in age-related GFR reduction is observed. The prevalence of high blood pressure and disorders of glucose and lipid metabolism also increases with age. These complications lead to a vicious circle that promotes renal function decline. Age-related decline in the rate of renal function is reportedly more rapid when GFR decreases at a younger age [11].

The prevalence of metabolic syndrome among adults in the USA is positively correlated with serum uric acid levels [7]. Metabolic syndrome, characterized by truncal obesity, hyperglycemia, elevated blood pressure, and insulin resistance, is recognized as a risk factor for kidney disease [8]. If obesity and metabolic syndrome have been caused at a younger age because of lifestyle factors such as poor dietary and exercise habits, lifestyle improvement may suppress renal function deterioration. However, the relationship between changes in renal function and various common risk factors including elevated blood pressure and abnormal blood glucose, lipid, and uric acid levels has not been studied in young and middle-aged persons.

Therefore, we performed the present study to evaluate the relationship between changes in renal function and common risk factors for CKD. Various confounding clinical factors influence changes in eGFR. To minimize the effect of such confounding factors, this study was limited to healthy middle-aged subjects aged ≤50 years who were not on medications for hypertension, diabetes mellitus, hyperlipidemia, or hyperuricemia and who had no proteinuria with ≥60 mL/min/1.73 m^2^ of eGFR.

## Subjects and Methods

This study was performed in accordance with the principles of the Declaration of Helsinki and was approved by the ethics committee of Murakami Memorial hospital. All subjects gave written informed consent for participation prior to the initiation of the study.

### Subjects and study design

This prospective observational study investigated the relationship between changes in renal function in healthy subjects aged ≤50 years and common risk factors for CKD. Apparently healthy persons who participated in a health screening program at our hospital from April 2009 to March 2010 were assessed for eligibility for this study (n = 5,728). We found a total of 3,188 participants aged ≤50 years with an eGFR of ≥60 mL/min/1.73 m^2^. The screening program (an interview regarding health status, routine physical examination, chest radiographic examination, electrocardiography, and laboratory tests for cardiovascular risk factors) revealed that 335 subjects among them had proteinuria or were on treatment for hypertension, diabetes, dyslipidemia, or hyperuricemia. Consequently, 2,853 subjects were enrolled in this study. The endpoint was the relationship between changes in eGFR and changes in body mass index (BMI), blood pressure, fasting plasma glucose (FPG), hemoglobin A_1c_ (HbA_1c_), lipids (including total cholesterol, LDL-cholesterol, HDL-cholesterol, and triglycerides), uric acid levels, and hemoglobin over 2 years. During the 2-year period, health screenings that included urine and blood tests on early morning specimens were conducted annually. CKD was defined as persistence of <60 mL/min/1.73 m^2^ eGFR for longer than 3 months. CKD was not evaluated by annual screening. The relationship between changes in eGFR, BMI, blood pressure, FPG, HbA_1c_, cholesterol, triglycerides, uric acid levels, and hemoglobin from baseline to 2-year follow-up was evaluated. The change in eGFR was set as a dependent variable, and changes in BMI, blood pressure, FPG, HbA_1c_, cholesterol, triglycerides, uric acid levels, and hemoglobin were used as independent variables for univariate analysis. In addition, the impact of longitudinal changes in each variable on the change in eGFR was assessed using multivariate analysis. HbA_1c_ was calculated as per the National Glycohemoglobin Standardization Program (NGSP) value according to the Japan Diabetes Society (JDS) guidelines: HbA_1c_ (NGSP) = 1.02×HbA_1c_ (JDS)+0.25 [12]. In the text and tables, HbA_1c_ (NGSP) values have been provided. Changes in variables (Δ) were calculated as the difference between the baseline value and the value at 2 years.

Blood pressure was measured after participants were seated in a chair for 5 min with their arms supported at heart level. Systolic and diastolic blood pressures were respectively recorded as the first and fifth Korotkoff sounds using a mercury sphygmomanometer. Three consecutive blood pressure measurements were taken, allowing 2 min between each measurement; the mean of the second and third measurements was recorded as blood pressure. eGFR was calculated using the Japanese Society of Nephrology formula [5].

### Statistical analysis

Data in the text and tables are expressed as mean ± SD. The normality of the distribution of variables was confirmed by checking a bell-shaped histogram. Subsequently, the differences between two variables with a normal distribution were compared using the paired *t*-test. The differences between two variables without a normal distribution were compared using the Wilcoxon signed rank tests. Nominal variables were compared using the McNemar's test. Simple and multiple linear regression analyses were performed to examine the relationship between changes in eGFR and changes in other variables over 2 years using the values at baseline and 2 years. *P* values of <0.05 were considered statistically significant.

## Results

### Changes in variables

**Figure 1:**
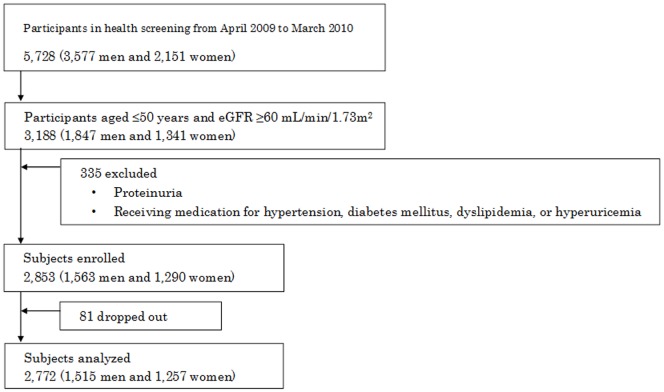
Disposition of the study population.

During 2-year follow-up, all except 81 subjects were assessed annually (Figure 1). No subject developed proteinuria or had to be started on medications for hypertension, diabetes, hyperlipidemia, or hyperuricemia.

**Figure 2:**
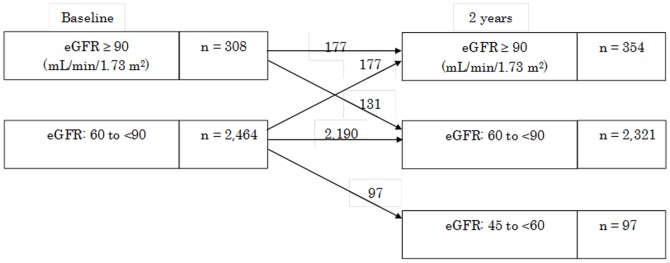
Change in eGFR over 2 years. eGFR was classified from stage 1 to stage 3a according to the CKD stage. Note that eGFR changed over 2 years.

During the study period, eGFR showed a significant decrease from 77.7±11.3 mL/min/1.73 m^2^ to 76.4±11.2 mL/min/1.73 m^2^ (Δ −1.25±8.56 mL/min/1.73 m^2^). BMI, systolic and diastolic blood pressure, FPG, HbA_1c_, total cholesterol, and HDL-cholesterol all showed a slight but significant increase (Table 1). Figure 2 shows changes in eGFR according to CKD classification between baseline and 2 years. eGFR did not always decline and was elevated in some subjects (Figure 2).

**Table 1:** Characteristics of subjects at baseline and 2 years.

Characteristic	Baseline	2 years	Δ	95% CI	*P* value
Gender (male/female)	1515/1257				
Age (years)	42.6±5.1	44.6±5.2			
Metabolic syndrome (n, %)	103, 3.7	119, 4.3			0.130 b
BMI (kg/m^2^)	22.21±3.15	22.34±3.21	0.14±0.95	0.10, 0.17	<0.001 a
Systolic BP (mmHg)	114.0±15.1	114.8±14.8	0.80±9.54	0.45, 1.16	<0.001 a
Diastolic BP (mmHg)	72.1±10.6	72.6±10.7	0.48±6.20	0.25, 0.72	<0.001 a
FPG (mg/dL)	93.4±10.9	93.9±11.7	0.56±7.74	0.27, 0.85	<0.001 a
HbA_1c_ (%)	4.89±0.37	4.94±0.37	0.04±0.22	0.04, 0.05	<0.001 a
Total cholesterol (mg/dL)	193.7±31.3	200.2±31.4	6.54±21.67	5.73, 7.36	<0.001 a
HDL-cholesterol (mg/dL)	57.0±13.5	59.6±16.0	2.56±8.25	2.25, 2.86	<0.001 a
LDL-cholesterol (mg/dL)	115.5±28.9	116.0±28.5	0.55±18.41	−0.13, 1.24	0.113 a
Triglycerides (mg/dL)	79.1±62.0	79.1±66.3	0.01±49.23	−1.82, 1.85	0.988 a
Uric acid (mg/dL)	4.84±1.38	4.88±1.35	0.04±0.65	0.02, 0.07	<0.001 a
Hb (g/dL)	13.84±1.76	13.82±1.72	−0.02±0.91	−0.06, 0.01	0.161 a
eGFR (mL/min/1.73 m^2^)	77.7±11.3	76.4±11.2	−1.25±8.57	−1.57, −0.93	<0.001 a

All values are expressed as mean ± SD. *P* values: a, paired *t*-test; b, McNemar's test comparing baseline with 2 years.

Abbreviations and symbols: Δ, change in the variable over 2 years; 95% CI, 95% confidence interval; BMI, body mass index; BP, blood pressure; FPG, fasting plasma glucose; HbA_1c_, hemoglobin A_1c_; eGFR, estimated glomerular filtration rate; HDL, high density lipoprotein; LDL, low density lipoprotein; Hb, hemoglobin.

### Results of linear regression analysis

Univariate analysis revealed that changes in FPG, total cholesterol, LDL-cholesterol, uric acid levels, and hemoglobin had a significant negative correlation with changes in eGFR (Table 2). To determine the independent contribution of each factor to the change in eGFR, a series of multivariate models based on risk factors for CKD were constructed. Model 1 included all factors, Model 3 was based on the significant factors in the univariate regression analysis, and Models 2 and 4 excluded total cholesterol because of multicolinearity in Models 1 and 3. Multivariate analyses using Model 3 revealed that changes in FPG, uric acid levels, and hemoglobin had a significant negative correlation with changes in eGFR, while BMI showed a positive correlation. The standardized regression coefficient (β) for changes in uric acid levels was the most negative among the significant variables (uric acid: −0.279; hemoglobin: −0.084; FPG: −0.080) (Table 3).

**Table 2:** Simple linear regression analysis of factors related to ΔeGFR.

Risk factors	ΔeGFR	
	β	*P* value
Baseline		
Male (vs. female)	−0.035	0.062
Age (years)	0.031	0.107
MetS (vs. no)	−0.006	0.770
Δ		
BMI (kg/m^2^)	0.002	0.934
Systolic BP (mmHg)	0.004	0.848
Diastolic BP (mmHg)	−0.036	0.061
FPG (mg/dL)	−0.080	<0.001
HbA_1c_ (%)	−0.025	0.184
Total cholesterol (mg/dL)	−0.081	<0.001
HDL-cholesterol (mg/dL)	−0.018	0.349
LDL-cholesterol (mg/dL)	−0.081	<0.001
Triglycerides (mg/dL)	−0.006	0.734
Uric acid (mg/dL)	−0.287	<0.001
Hb (g/dL)	−0.148	<0.001

All values are expressed as mean ± SD.

Abbreviations and symbols: eGFR, estimated glomerular filtration rate; Δ, change in the variable over 2 years; β, standardized regression coefficient; MetS, metabolic syndrome; BMI, body mass index; BP, blood pressure; FPG, fasting plasma glucose; HbA_1c_, hemoglobin A_1c_; HDL, high density lipoprotein; LDL, low density lipoprotein; Hb, hemoglobin.

**Table 3:** Multiple regression models for factors related to ΔeGFR.

	ΔeGFR							
Risk factors	Model 1 (R^2^ = 0.105)		Model 2 (R^2^ = 0.102)		Model 3 (R^2^ = 0.097)		Model 4 (R^2^ = 0.94)	
	β	*P* value	β	*P* value	β	*P* value	β	*P* value
Δ								
BMI (kg/m^2^)	0.056	0.007	0.056	0.006				
Systolic BP (mmHg)	0.046	0.067	0.047	0.060				
Diastolic BP (mmHg)	−0.044	0.077	−0.046	0.068				
FPG (mg/dL)	−0.084	<0.001	−0.080	<0.001	−0.088	<0.001	−0.081	<0.001
HbA_1c_ (%)	−0.023	0.249	−0.021	0.290				
Total cholesterol (mg/dL)	0.142	0.014			0.111	0.007		
HDL-cholesterol (mg/dL)	−0.022	0.388	0.025	0.213				
LDL-cholesterol (mg/dL)	−0.147	0.006	−0.022	0.258	−0.110	0.006	−0.012	0.532
Triglycerides (mg/dL)	−0.012	0.625	0.022	0.255				
Uric acid (mg/dL)	−0.284	<0.001	−0.279	<0.001	−0.273	<0.001	−0.269	<0.001
Hb (g/dL)	−0.087	<0.001	−0.084	<0.001	−0.084	<0.001	−0.073	<0.001

Model 1: all variables, Model 2: all variables except total cholesterol, Model 3: significant variables in univariate analysis, Model 4: significant variables in univariate analysis except total cholesterol.

Abbreviations and symbols: eGFR, estimated glomerular filtration rate; Δ, change in the variable over 2 years; β, standardized regression coefficient; BMI, body mass index; BP, blood pressure; FPG, fasting plasma glucose; HbA_1c_, hemoglobin A_1c_; HDL, high density lipoprotein; LDL, low density lipoprotein; Hb, hemoglobin.

### Prevalence of hyperuricemia

The influence of hyperuricemia on changes in eGFR was also analyzed. At baseline, hyperuricemia (serum uric acid levels ≥7 mg/dL in men and ≥6 mg/dL in women) was found in 12.6% of men and 1.0% of women. After 2 years, fewer subjects had hyperuricemia than at baseline (Table 4). Almost all changes in serum uric acid levels were within the normal range.

**Table 4:** Prevalence of hyperuricemia (male, n = 1,515; female, n = 1,257).

Gender	Baseline	2 years
Male, n (%)	191 (12.6)	194 (12.8)
Female, n (%)	12 (1.0)	12 (1.0)

Hyperuricemia: serum uric acid ≥7 mg/dL in males and ≥6 mg/dL in females.

## Discussion

This prospective observational study was the first to evaluate the relationship between changes in eGFR and changes in variables related to renal function over 2 years in healthy subjects aged ≤50 years. This study demonstrated that changes in the serum uric acid levels that were largely within the normal range were a sensitive indicator of changes in eGFR and thus changes in renal function in healthy middle-aged subjects. During the 2-year follow-up, eGFR did not always change unidirectionally (Figure 2). An increase in eGFR was associated with a decline in serum uric acid levels, while a decrease in eGFR was associated with elevation of serum uric acid levels. eGFR was calculated according to patient age and serum creatinine levels; it decreases with aging and an increase in serum creatinine levels. In this study, the changes in eGFR over only 2 years were more strongly influenced by the decrease in serum creatinine levels than the increase in age. Thus, decline in serum creatinine levels with age generally meant elevation of eGFR so that renal function was maintained. However, changes in eGFR in some of the present subjects may also represent physiological changes in renal function; the observation period was too short to determine the direction of change in eGFR in some cases. There was a significant association between changes in hemoglobin and those in eGFR (table 1, 2), although hemoglobin did not change significantly in this time frame. These results showed that subjects with positive changes in hemoglobin which were not significant had larger negative changes in eGFR in all subjects, and that a very slight hemoglobin decline within the normal range was associated with elevation of eGFR. Elevation of eGFR may be related to a decrease in hemoglobin because of the alteration of fluid balance. Whether the change in serum uric acid levels within the normal range has any clinical importance has been unclear. Therefore, these changes in eGFR and other variables over 2 years are so small but statistically significant that they are clinically meaningless.

A study by Bellomo et al. [13] in healthy normotensive individuals showed that serum uric acid levels were associated with the risk of eGFR decline over 5 years. They analyzed changes in eGFR as a continuous variable rather than as a variable in the incidence of stage 3 CKD. The adjusted hazard ratio for a decrease in eGFR by 10 mL/min/1.73 m^2^ was 1.23 (95% confidence interval: 1.09, 1.39) for every 1.2 mg/dL increase in the serum uric acid level. Serum uric acid levels showed a slight, but significant, relationship with eGFR decline using multiple regression analysis (β, women: −0.17; β, men: −0.15). More recently, Iseki et al. [14] demonstrated that an increase in the serum uric acid level with or without hyperuricemia over a 10-year period was more closely related to eGFR decline than the baseline serum uric acid level or the presence of diabetes, and that an increase in uric acid levels within the normal range (N/N) was more closely associated with eGFR decline than the change in uric acid levels from normouricemia to hyperuricemia (N/H) or any variation within the hyperuricemic range (H/H) (decrease in eGFR per 1 mg/dL increase of serum uric acid levels was 4.19 mL/min/1.73 m^2^ for N/N, 2.36 mL/min/1.73 m^2^ for N/H, and 2.01 mL/min/1.73 m^2^ for H/H). These results suggest that changes in eGFR related to the change in serum uric acid levels over several years may represent physiological variations, but it may also be an early indicator of renal dysfunction in a healthy middle-aged population.

Linear regression analysis revealed significant β values for changes in serum uric acid levels, hemoglobin, FPG, and BMI despite the relatively low coefficient of determination (R^2^ = 0.102). As R^2^ seemed to be relatively low because regression analysis included confounding variables for the change in eGFR, the change in serum uric acid levels (which showed the most negative β value of −0.279) was not sufficient to completely explain the change in eGFR. In other words, low R^2^ values revealed that the change in eGFR was influenced by many confounding factors. However, this study demonstrated that the change in serum uric acid levels was the most sensitive factor for the change in eGFR among those evaluated in this population.

The association between elevated serum uric acid levels at baseline and progression of CKD has been reported previously [13], [15]–[18]. A two-year study of Japanese residents of Okinawa revealed that a higher baseline serum uric acid level was associated with elevation of serum creatinine levels over 2 years [15]. Another Japanese study of healthy subjects with normal renal function and no proteinuria, who were not receiving medications for hypertension, diabetes, or hyperlipidemia, and who underwent annual health screening demonstrated that the baseline serum uric acid level was an independent predictor of future development of CKD (eGFR <60 mL/min/1.73 m^2^) after a median of 4.6 years [16]. Moreover, an association has been reported between elevated serum uric acid levels and metabolic syndrome, which are known risk factors for CKD [7], [9], [10].

The current classification of hyperuricemia is based on the concept that this condition results from either overproduction of urate due to a metabolic disorder, underexcretion due to abnormal renal urate transport activity, or a combination of the two [19]. Underexcretion of urate was widely considered to be the main cause of elevation of the serum uric acid level associated with renal function decline; therefore, an elevated serum uric acid level has been found to be a marker for CKD rather than as a cause of CKD [20], [21]. However, recent epidemiological studies have demonstrated that elevated serum uric acid levels are associated with the progression of renal disease in humans [13], [15]–[18] and can accelerate renal disease in animals [22]–[24]. A 12-month epidemiological study of general population without renal dysfunction revealed that serum uric acid levels were positively correlated with albuminuria, suggesting glomerular damage [25]. Animal studies have indicated that an elevated serum uric acid level may be directly toxic to the kidney [22]–[24]. Mild hyperuricemia may cause direct renal toxicity in rats, which was manifested by renal vasoconstriction, systemic hypertension, and tubulointerstitial injury via a crystal-independent mechanism [22]. Uric acid may have these effects through the inhibition of endothelial nitric oxide bioavailability, activation of the renin–angiotensin system, and/or a direct influence on endothelial and vascular smooth muscle cells [26]–[28]. This hypothesis was clinically supported by the report that elevation of uric acid levels was a risk factor for hypertension [29]–[31]. An ultrasonographic study of flow-mediated dilatation demonstrated that elevation of uric acid levels was an independent predictor of endothelial dysfunction in patients with CKD [32]. In addition, uric acid has been found to promote oxidative stress through the renin–angiotensin system in cultured human endothelial cells [33]. Two small, short-term, single-center studies have shown improved blood pressure control and the slowing of the progression of CKD after serum uric acid level reduction with allopurinol [34], [35]. The association of uric acid with CKD in the above reports has suggested that uric acid may be directly toxic to the kidney, and that elevation of uric acid levels may exacerbate other factors that promote CKD such as hypertension. Uric acid may also be a risk factor for metabolic syndrome. Moreover, elevation of serum uric acid levels may be an independent risk factor for renal injury based on the above reports.

This study showed that elevation of BMI and FPG was associated with a decrease in eGFR. These have both been known as risk factors for CKD [2], [6], [36].

This study had some limitations. First, the subjects were limited to participants undergoing health screening at our center, suggesting that they may not be representative of the healthy Japanese population ≤50 years old. Second, the 2-year study period was too small to meaningfully assess changes in renal function because serum creatinine levels were only measured once a year. The impact of serum creatinine levels may be more than age on changes in eGFR (calculated from age and serum creatinine levels) over the 2-year period. Interpretation of serum creatinine levels can also be directly influenced by muscle mass, exercise, and fluid balance disorders such as dehydration. Third, the eGFR changed at a statistically significant level between baseline and 2-year follow-up values. Hemoglobin did not change significantly in this time frame. Nevertheless, there was a significant association between changes in hemoglobin and those in eGFR (table 1, 2). These changes could be within the respective biologic variation. A longer epidemiological study about changes in eGFR and other variables is needed for health screening. Fourth, some subjects had low eGFRs at baseline. A high serum creatinine level was previously reported to be >8.0% in a community-based study [37]. Finally, this study did not evaluate urate production or the influence of age on serum uric acid levels. In particular, dietary intake, including the amount and types of alcohol, should be evaluated as a confounding variable that can influence uric acid production. It has been reported that hyperuricemia was negatively correlated with age in men [38], [39]. Thus, changes in the serum uric acid level may be somewhat augmented by age.

In conclusion, the changes in eGFR and other variables over 2 years were small and could be within expected biologic variation. A longer observational study is needed to elucidate whether FPG, serum uric acid and hemoglobin represent the earliest markers of eGFR decline.
